# Physical, Chemical, Microstructural and Rheological Properties of Reactive Terpolymer-Modified Bitumen

**DOI:** 10.3390/ma12060921

**Published:** 2019-03-20

**Authors:** Tacettin Geçkil

**Affiliations:** Department of Civil Engineering, Faculty of Engineering, Inonu University, 44280 Malatya, Turkey; tacettin.geckil@inonu.edu.tr; Tel.: +90-532-581-1889

**Keywords:** bitumen, reactive terpolymer, aging, microstructure, stiffness, rheological property

## Abstract

In this study, the change in the physical, chemical, microstructural, and rheological properties of a road bitumen modified with reactive terpolymer (Elvaloy^®^RET) was investigated. For this purpose, four different Elvaloy^®^RET percentages (0%, 0.4%, 0.8%, and 1.2% by wt.) were mixed with B 160/220 bitumen. Firstly, the obtained samples were subjected to a short and long-term aging process using a rotating thin-film oven test and pressurized aging vessel, respectively. Then, the engineering characteristics of the samples were investigated using X-ray diffraction, scanning electron microscopy, and conventional and Superpave bitumen tests. The results showed that Elvaloy^®^RET reacted with the main macromolecular groups in the bitumen, and that the modified bitumens behaved as a homogenous single phase structure as a result of this reaction. Conventional tests showed that Elvaloy^®^RET-modified bitumens were much harder than pure bitumen and had better aging resistance. Furthermore, the penetration classes of these bitumens changed and their temperature sensitivities decreased significantly. Rheological tests showed that the Elvaloy^®^RET additive increased the resistance of the bitumen against fatigue cracking and particularly permanent deformation. Moreover, the high temperature performance class of the bitumen also increased. However, parallel to the hardening of the bitumen, a reduction in its resistance to cracking at low temperature was observed.

## 1. Introduction

Some conditions such as different environment, climate, heavy traffic, and tire pressure lead to various deteriorations in asphalt pavements. The most common deteriorations in pavements are the permanent deformations at high temperatures, fatigue cracks at intermediate temperatures, and thermal cracks at low temperatures [1,2,3,4,5,6,7,8]. Nowadays, asphalt binders are modified with various additives in order to prevent or reduce these deteriorations. Because the performance of an asphalt pavement is significantly affected by the low and high temperature performance of binders. For this reason, many natural and chemical additives have been used in recent years in order to improve some properties of conventional bitumens such as aging stability, thermal sensitivity, elastic behavior, and cracking resistance [5,9,10,11,12].

Recently, the use of polymer-based additives has been greatly increased in bitumen modification. In particular, two main polymer types, elastomers, and plastomers have been widely used in studies. The plastomers have generally been used to improve the elastic properties of binders and to increase the permanent deformation resistance of the pavements. However, the elastomers have been used to improve the fatigue and low temperature crack resistance of the binders [5,13,14,15,16,17].

In this study, Reactive Terpolymer (Elvaloy^®^RET), an elastomeric polymer, has been used as a bitumen additive to determine its effects on the engineering properties of a soft consistency bitumen.

Reactive terpolymers are the polymers containing functional groups which can form chemical bonds with some bitumen components. These polymers form a network around the asphaltenes in the bitumen to form an integral compound and produce a chemical reaction with the bitumen [17,18,19,20,21,22,23]. However, when these chemical bonds occur, a polymer bitumen gel may be formed which in this case does not have melting and dissolving properties and is completely useless [18,19,22]. Therefore, it is desirable that the amount of terpolymer to be used in its modification has an upper limit to remain below the chemical gel point of the network [18,19,21]. In some studies, this limit is considered to be 2–2.5% by weight, although it is stated that it may be less than 1% [19,22]. According to some researchers, much better results can be achieved with a bitumen containing a less reactive terpolymer group. Furthermore, the use of a higher content of terpolymer has been considered an economic disadvantage. It has also been reported in the studies that the use of terpolymers alone as a modifier is not ideal, because it may have a negative impact on the performance properties of binder due to the gelation [18,19,20,22]. When reactive terpolymers are used in bitumen modification, a certain amount of Polyphosphoric Acid (PPA) is usually added as a catalyst to accelerate the chemical reaction between bitumen and polymer in the mixture [17,18,20,23,24]. When PPA is used in the bitumen–polymer mixture, the amount of polymer needed for the modification is reduced and the performance characteristics of the modified bitumen, such as permanent deformation, aging and fatigue resistance, increase positively [17,18,19,20,21,24].

In some previous studies, it has been stated that the use of terpolymer in bitumen modification increases the viscosity and softening point of binder and decreases its ductility and penetration [25,26,27,28,29,30,31,32]. In one of these studies, bitumens with different physical properties were modified with Elvaloy^®^, and as a result, an increase of about 353% was observed in the viscosity of these bitumens [30]. In a study using 0%, 1.5% and 2.0% Elvaloy^®^ according to the weight of bitumen, it was observed that the sensitivity of asphalt mixtures to moisture damage decreased significantly with the increase of Elvaloy^®^. In the study, it was also determined that Elvaloy^®^ had a positive effect on the rutting performance of asphalt mixtures [33]. In addition, the results of another study showed that a binder modified by reactive terpolymer improved the resistance to permanent deformation at high temperatures [34]. In another study, which was carried out by using Elvaloy in bitumen modification, the optimum ratio of Elvaloy additive was determined as 1.0% in terms of rutting resistance and high and low temperature. In the study, this situation was explained by the polymer-linked binder formed by the chemical reaction between Elvaloy and binder. The study also revealed that the minimum mass loss among modified bitumens occurs in blends containing 1% Elvaloy [32]. In many studies conducted to determine the mass loss or aging resistance of terpolymer-modified bitumens, it has been found that Elvaloy affects the aging resistance of bitumen very positively [19,20,22,28,31]. In studies, this was explained as a result of the chemical reaction between the reactive terpolymer and the carboxylic acid groups present in the bitumen [18,20,22,32]. The studies showed that the internal structure of the modified bitumen exhibits a homogenous distribution as a result of the reaction between Elvaloy^®^RET and binder, and consequently the problem of the decomposition which may occur through transportation and storage has disappeared [3,14,17,30,35].

Furthermore, many studies have shown that the addition of Terpolymer in the bitumen causes an increase in the complex module of the modified bitumen, but a decrease in its phase angle. This indicates that Elvaloy^®^-modified bitumens provide a general improvement in the rutting resistance of the pavements [17,18,33,35,36,37]. In a study in which rheological properties of bitumens modified with different polymers were investigated at low temperatures, it was found that the cracking resistance of the bitumens containing Elvaloy^®^RET was lower than the others [3]. However, in some studies it has been noted that the use of terpolymer in bitumen modification increases its high temperature performance without significantly damaging the low temperature performance of modified bitumen [31,32,37]. In a study using B 100/150 bitumen and different Elvaloy ratios, the effects of Elvaloy^®^RET on the physical and rheological properties of the bitumen were investigated. The results of the study showed that due to the increase in the amount of additive, the hardness of the modified bitumens increased and the temperature sensitivity decreased. Furthermore, although the low temperature performances of the modified bitumens were relatively low, their high temperature performances increased considerably [38].

When the studies were examined, it was observed that terpolymers were used alone or in combination with PPA in two different ways in bitumen modification. It was also seen that the additive percentages of terpolymer were higher (1–6%) when it was used alone [26,27,28,29,31,32,34], but were lower (0.4–1.6%) when it was used with PPA [14,17,18,20,24,25,28,30,38]. Moreover, the terpolymers were generally used in a single percentage and in combination with another polymer [17,18,20,24,28,30,31,34]. Although there have been studies using different percentages of terpolymer in the modification, PPA has been used in only two of them [25,38]. In one of these studies, physical and chemical characterization of modified bitumen were examined [25], while in the other study its physical and rheological properties were investigated [38]. As a result, it was seen that the use of Elvaloy^®^RET in different percentages and in combination with PPA was very limited, and a limited number of experiments were performed to determine the properties of Elvaloy-modified bitumen.

The aim of this study was to understand the effects of Elvaloy^®^ 5160 additive on the physical, chemical, micro-structural, and rheological properties of B 160/220 bitumen with all aspects. In order to determine the characteristics of Elvaloy^®^-modified bitumens, tests such as X-Ray Diffraction (XRD), Scanning Electron Microscopy (SEM), penetration, softening point, ductility, Rotational Viscosity (RV), Dynamic Shear Rheometer (DSR), and Bending Beam Rheometer (BBR) were performed.

## 2. Materials and Methods

### 2.1. Materials

#### 2.1.1. Neat Bitumen

The neat bitumen used for this study is an asphalt binder with B 160/220 penetration grade whose physical properties are presented in Table 1. This was supplied from Turkish Petroleum Refineries Corporation (TUPRAS, İzmit, Turkey) and is a commonly used binder in Turkey.

#### 2.1.2. Reactive Terpolymer

In the study, the reactive terpolymer (Elvaloy^®^ 5160) supplied by DuPont Company (Wilmington, DE, USA) was used as the modifier. Its characteristics are presented in Table 2.

It enters a chemical reaction with bitumen at high temperatures and forms a covalent bond with the bitumen molecule and does not separate [28]. As a result of this reaction, a netting structure formed around the asphaltenes increases the stiffness and elasticity of the bitumen [14,20,28]. This is the result of the reaction of the reactive terpolymer with the groups of asphaltenes in bitumen [25,30,35].

#### 2.1.3. Poly Phosphoric Acid (PPA)

In order to accelerate the chemical reaction between bitumen and Elvaloy^®^RET, PPA was used as a catalyst. PPA was obtained from the department of chemical engineering at Inonu University (Malatya, Turkey). The properties of PPA are given in Table 3.

**Figure 1:**
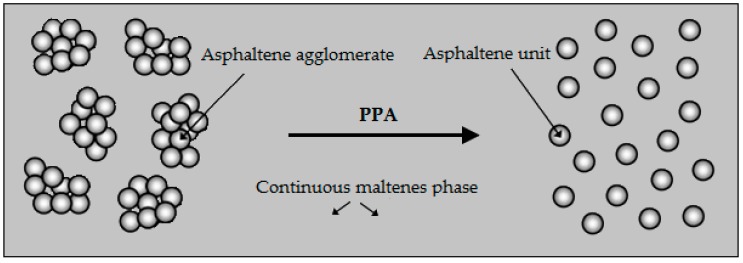
The function of PPA in asphalt binder [39].

PPA breaks the asphaltene agglomerates by reacting with many of the functional groups present in the bitumen and provides a better distribution of asphaltene in the molten phase as shown in Figure 1. As a result, it contributes to the elastic behaviour of the bitumen by converting them into individual particles [39].

#### 2.1.4. Preparation of Binder Samples

Elvaloy^®^RET-modified bitumens were prepared according to the instructions given in the asphalt modification manual of DuPont. Additive percentages were selected taking into consideration the limit values proposed by the manufacturer for bitumen modification [35]. Mixtures containing more than 1.2% Elvaloy^®^RET were not used in bitumen modification because the chemical reaction that continued throughout the mixing process produced an insoluble gelling in the mixture containing 1.4% Elvaloy^®^. For this reason, modified bitumens were prepared by adding 0%, 0.4%, 0.8%, and 1.2% Elvaloy^®^RET to the neat bitumen. All modified bitumens were prepared by mixing in a mixer at a temperature of 185 °C for 120 min at a rotational speed of 500 rpm. Poly phosphoric acid was added as a catalyst in a proportion of 0.3% by weight of neat bitumen to the prepared mixtures, and the mixing process was continued for an additional 30 min. Once the mixing process of each sample was completed, it was cured at 185 °C for 90 min. This provided the completion of the chemical reaction between bitumen and Elvaloy^®^RET, which allowed the formation of permanently modified binders.

The engineering properties of the bitumen samples containing the neat and three different Elvaloy^®^RET percentages were investigated using conventional and Superpave bitumen tests. These bitumens used in the study were coded as B, B + 0.4R, B + 0.8R and B + 1.2R respectively.

### 2.2. Experimental Methods

#### 2.2.1. Short and Long Term Aging Methods of Binders

In the study, according to the ASTM D2872 procedure, the amount of aging occurring in the binders during the mixing and construction process, that is, the short-term aging of the binders was determined using the thin film oven test (RTFOT). In the test, glass bottles, each containing 35 g of asphalt binder, were placed in a rack rotating at a constant speed (15 rpm) in an oven at 163 °C; and exposed to air flowing at a constant rate of 4000 mL/min for 80 min. Long-term aging in the binders, i.e., service aging in the binders in a period of 8–10 years following the pavement construction, was carried out by applying the pressure aging vessel (PAV) process according to the ASTM D 6521 standard. In the test, RTFOT-aged bitumen samples (50 g) were aged by exposure to pressurized (2.10 MPa) air at 100 °C for 20 h. The residues of the bitumen samples obtained from the PAV were degassed by being placed in a vacuum oven immediately after being subjected to conditioning.

#### 2.2.2. X-ray Diffraction (XRD) Test

**Figure 2:**
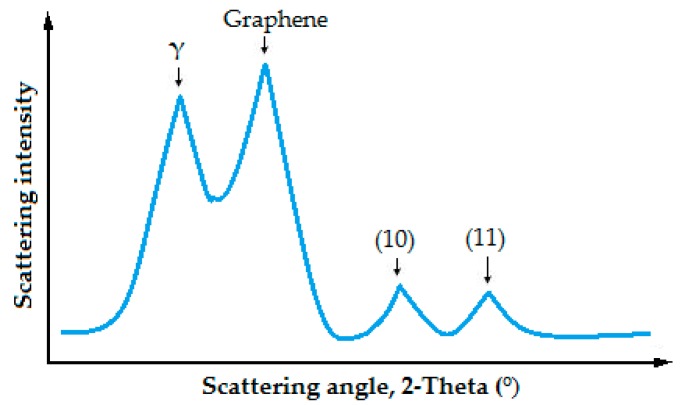
XRD pattern of a bitumen sample [40].

XRD (Giegerflex D-Max/B, Rigaku, The Woodlands, TX, USA) is a non-destructive and versatile analysis method used to identify crystalline phases in solid materials and to analyze the structural properties of these phases. XRD is widely used to determine the crystallite parameters of modified bitumens and provides quantitative intensity curves according to the peak intensity and position of the structural parameters in the bitumen sample (Figure 2).

In the XRD model of a bitumen, there are basically four peaks resulting from the scattering of X-rays from the molecular structure of the bitumen. The γ peak refers to the packing distance of saturated structures originating from X-rays scattered by aliphatic chains. The graphene peak consists of diffraction of X-ray due to the stacks of aromatic molecules. The (10) and (11) peaks in the pattern originate from the in-plane structure of the aromatics. The long, sharp and narrow XRD pattern peaks generally indicate that the sample has a high degree of crystal structure [40].

#### 2.2.3. Scanning Electron Microscopy (SEM) Test

Scanning electron microscope (SEM, EVO 50, Zeiss, Oberkochen, Germany) is a versatile characterization tool that allows the observation and analysis of micromorphology of materials. SEM is the most valuable method used to study the phase morphology of the polymer-modified bitumen, since it allows the monitoring of the structure and homogeneity in the material. SEM images can be used to provide information on the dispersion of the polymer-rich phase in the modified binder, to provide an understanding of the bitumen-polymer compatibility, to find the optimum polymer content, and to reveal the damage resulting from the aging process. In SEM images, the bitumen-rich phase appears dark or black, and the polymer-rich phase appears light [41].

#### 2.2.4. Conventional Binder Tests

In order to determine the physical characteristics of the neat and modified binders, they were subjected to conventional tests such as penetration, softening point, and ductility in accordance with ASTM D36, ASTM D5, and ASTM D113 standards, respectively. Furthermore, by using these test results, the penetration index (*PI*) value, which is accepted as a measure of the temperature sensitivity of the binders, was calculated using the following equation [42]:

(1) $$PI=\frac{1952-500\times \log({Pen}_{25})-20\times SP}{50\times \log({Pen}_{25})-SP-120}$$

where, *Pen*_25_ is penetration at 25 °C, and *SP* is the softening point temperature of the binder.

If the *PI* value of a bitumen is low, the temperature sensitivity is high, and if the *PI* value is high, the temperature sensitivity is considered to be low. When using a bitumen with a higher *PI* value in an asphalt mixture, the mixture becomes more resistant to the rutting and thermal cracking [43].

#### 2.2.5. Rotational Viscosity (RV) Test

The RV test (DV-III, Brookfield, Harlow, UK) was carried out in accordance with ASTM D 4402 standard procedures to determine the flow properties of binders at high temperatures as a measure of workability and pumpability. In order to determine the mixing and compaction temperatures of asphalt binders, it was proposed by the Asphalt Institute that viscosity measurements should be taken at 135 °C and 165 °C, respectively. However, it has been indicated that the RV value measured at 135 °C of the binder should not exceed 3000 cP. For mixing and compaction temperatures, it has been proposed to use temperature ranges corresponding to the viscosity limits of 170 ± 20 cP and 280 ± 30 cP respectively [44,45].

#### 2.2.6. Dynamic Shear Rheometer (DSR) Test

The DSR test (Bohlin DSR II, Malvern Panalytical Ltd., Malvern, UK) is used to determine the phase angle (δ) and the complex modulus (G*) to measure the visco-elastic properties and performance levels of the bitumens at intermediate and high temperatures. With these parameters obtained at different temperatures, it is possible to estimate the resistance of asphalt pavements to fatigue cracks and permanent deformations.

The visco-elastic behaviors of the binders at intermediate and high temperatures were determined in the stress control mode at the frequency of 10 rad/s using a Bohlin DSR II rheometer according to ASTM D7175 standard. In order to evaluate the effects of Elvaloy^®^RET on performance of bitumens at high service temperatures, the DSR test was performed on the bitumens before and after the RTFOT aging process. The test was performed at high temperatures from 52 °C to 76 °C using samples with 1.0 mm thickness and 25.0 mm diameter. As a result of the test, the rutting parameters (G*/sin δ) and performance levels of binders were determined. Moreover, PAV-aged bitumens were tested at 19, 22, 25, and 28 °C under shear loading using 2.0-mm thickness and 8.0-mm diameter specimens to determine their performance at intermediate service temperatures, and fatigue parameters (G*.sin δ) were determined.

According to the Superpave specification, a minimum of 1.00 kPa for original un-aged binders and a minimum of 2.20 kPa for RTFOT-aged binders are given as limit values (both values are G*/sin δ). PAV-aged binder samples have a maximum specification of 5000 kPa for G*.sin δ. Higher G* and lower δ values in terms of permanent deformation resistance, but lower G* and higher δ values in terms of fatigue cracking resistance are desired [44,45].

#### 2.2.7. Bending Beam Rheometer (BBR) Test

The BBR test (BBR 2, ATS, Butler, PA, USA) has been developed to assess the thermal cracking resistance and stiffness of asphalt binders at very low air temperatures. With this test, the creep stiffness (S), which is an indicator of the binder stiffness, and the m-value which is a measure of the change in binder hardness, are determined. Superpave specified 300 MPa maximum for S and a 0.300 minimum m-value [44,45].

PAV-aged samples were subjected to this test according to ASTM D6648 standard to determine the effects of Elvaloy^®^RET on the low temperature performance properties of the binders. The test was carried out using a BBR device at −16 °C, −22 °C, and −28 °C. The performance levels of the binders at low temperatures were determined with the aid of creep stiffness (S) and creep rate (m-value) parameters were obtained as a result of the test. 

## 3. Results and Discussion

### 3.1. X-ray Diffraction

The morphology of Elvaloy^®^RET-modified bitumens was determined using the XRD characterization techniques. The X-ray diffraction patterns were obtained by a Rigaku Giegerflex D-Max/B powder diffractometer with CuKα radiation. Diffraction patterns (2θ = 2–80°) were recorded at room temperature with a step of 0.02°. The crystallographic structure of Elvaloy^®^RET, its presence in the bitumen blend, and its level of dispersion were evaluated using the patterns. 

**Figure 3:**
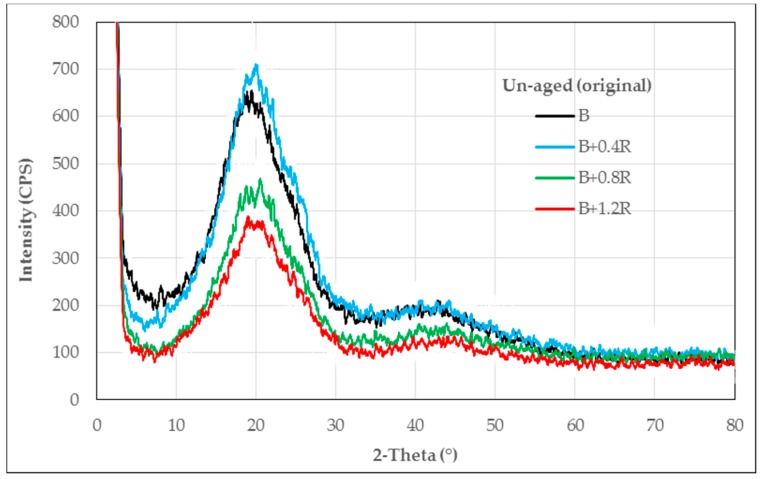
XRD patterns of the neat and Elvaloy^®^RET-modified bitumens.

**Figure 4:**
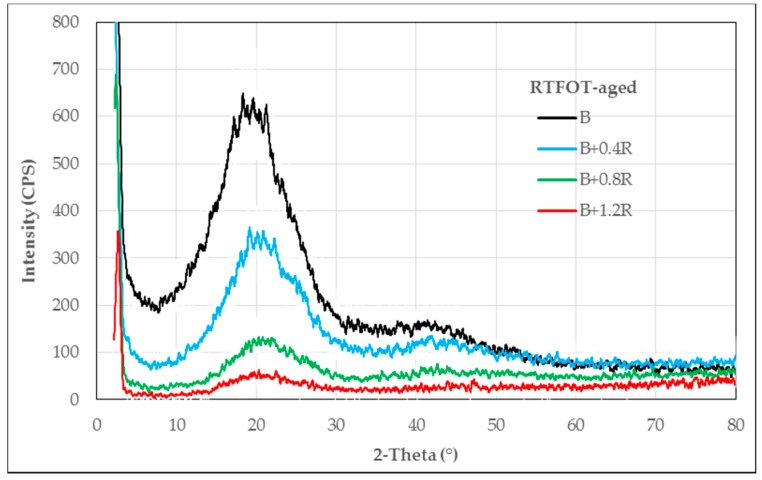
XRD patterns of RTFOT-aged bitumens.

**Figure 5:**
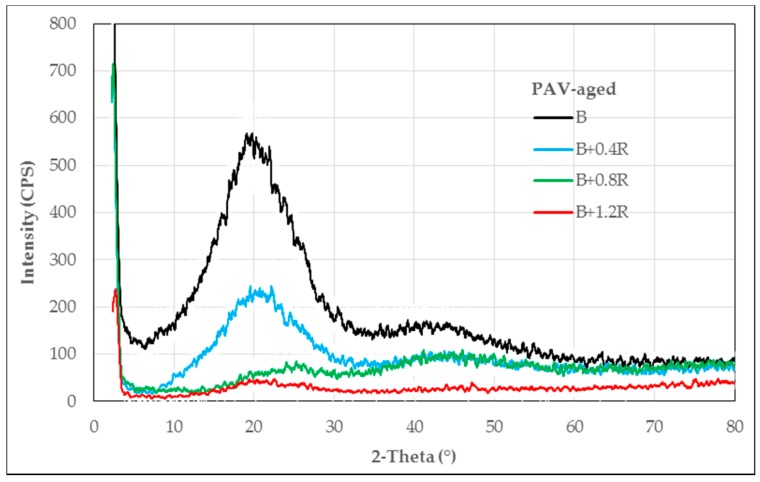
XRD patterns of PAV-aged bitumens.

The XRD patterns obtained from neat and modified bitumens are given in Figure 3, Figure 4 and Figure 5, respectively, for un-aged (original), RTFOT-aged, and PAV-aged samples.

As seen in Figure 3, the XRD traces of the neat bitumen sample used in the experiment are consistent with those given in the literature. The structure is generally a macromolecular single amorphous structure. The wide band of 2-theta between 7.5 and 32.5 indicates that the structure is amorphous. However, the height of the peak shows that there are crystal shadows in the structure. At the point where the 2-theta is about 40, there is a relatively small amorphous phase in the structure. In summary, the neat bitumen has a macromolecular structure in two separate amorphous phases. The amorphous phase at the point where 2-theta is about 40 is located within the main macromolecular structure.

When 0.4% Elvaloy^®^RET is added to bitumen, there is no significant change in the macromolecular structure of the modified bitumen. However, when the amount of terpolymer in the bitumen blend increases, the number of counts decreases. That is, when the bitumen and Elvaloy^®^RET react chemically, the terpolymer group in the macromolecular structure absorbs X-rays. As a result, although the number of counts was 705 at the point where 2-theta was 20 for pure bitumen, these values were 435 and 300 respectively when 0.8% and 1.2% Elvaloy^®^RET were added to the bitumen. However, there is no significant change in the amorphous structure area around the point where the 2-theta is 40. This indicates that Elvaloy^®^RET interacts with the main macromolecular groups in the bitumen and exhibits a uniform distribution within the bitumen blend.

As seen in Figure 4, when the XRD data of RTFOT-aged bitumen samples were examined, it was seen that the crystallinity in the structure decreased with the addition of Elvaloy^®^RET into bitumen. Under short-term aging conditions, with the increase in the terpolymer, it is seen that the crystalline components in the mixture disappear and the amorphous structure of the terpolymer stands out. In other words, the amorphous structure of the products formed as a result of the bitumen-terpolymer reaction dominated the bitumen blend.

As seen in Figure 5, in PAV-aged samples, the gaps between the components in the macromolecular structure have been closed by the effect of temperature and high pressure. In the region where the 2-teta was between 7.5–32.5, the semi-crystalline region was transformed into a completely amorphous structure, while the amorphous structure at the point where the 2-teta was about 40 was enlarged and spread according to the neat bitumen. However, when the Elvaloy^®^RET ratio was 1.2%, the structure was transformed into a completely amorphous structure as a result of the macromolecular arrangement with the effect of pressure and temperature.

### 3.2. Scanning Electron Microscopy

SEM was used to investigate the microstructure of Elvaloy^®^RET-modified bitumens, to determine the distribution state of Elvaloy^®^RET in the neat bitumen, and to characterize the structure of the continuous and discontinuous phase. Surface imaging of all bitumen samples was performed using SEM (Zeiss EVO 50 Model) with a magnification of 1.0 K, electron acceleration voltage of 20 kV, and zooming of 20 µm.

**Figure 6:**
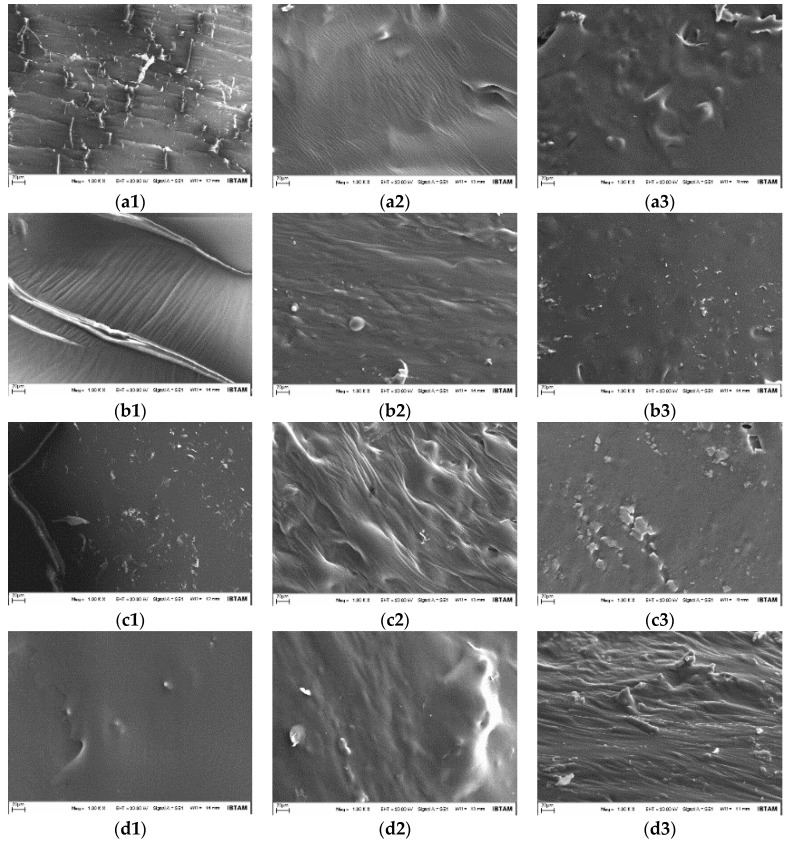
SEM images (1.0 K magnification, 20 kV voltage and 20 µm zooming) of bitumen samples before and after aging process: (**a**) B; (**b**) B + 0.4R; (**c**) B + 0.8R; (**d**) B + 1.2R, for (**1**) un-aged (original); (**2**) RTFOT-aged; and (**3**) PAV-aged.

SEM images obtained from neat and modified bitumens are shown in Figure 6 for un-aged (original), RTFOT-aged, and PAV-aged samples.

As seen in Figure 6(a1,b1,c1,d1), the SEM image of the pure bitumen coincides with the literature and it is seen that its structure is a homogeneous single phase. Considering the Elvaloy^®^RET-bitumen interaction in the modified bitumens, it is seen that homogeneous structure is preserved at all three ratios. Elvaloy^®^RET is known to be chemically reacted with bitumen. Therefore, the Elvaloy^®^RET- bitumen mixture behaves as a single component. The XRD results also support this data, and the XRD peak intensity decreases due to the increased amount of Elvaloy^®^RET.

Figure 6(a2,a3,b2,b3,c2,c3,d2,d3) shows that, as a result of the aging, the pure and modified- bitumen samples were transformed into an amorphous structure. Considering the aging conditions, it can be stated that the fraction missing in the binder is the component giving a crystalline feature to the structure. As the amount of Elvaloy^®^RET in the bitumen increases, the decline of mass loss and the increase in the amorphous structure indicates that the terpolymer forms a bond with the amorphous groups rather than the crystalline groups. In the SEM images, structural changes were observed on the surface of modified bitumens compared to pure bitumen. It is thought that this is due to the fact that the molecular groups that leave the bitumen structure due to aging conditions change the surface geometry.

In conclusion, in the SEM images of short and long-term aged samples, homogeneity in the structure was clearly seen in terms of surface morphology. This was expected due to the bitumen-Elvaloy^®^RET reaction, and the XRD findings also supported this result. As a result of this reaction, the structure of the bitumen has become homogeneous and due to the removal of small groups of crystals as a result of aging, a homogenous amorphous macromolecular structure was formed.

### 3.3. Conventional Asphalt Tests

**Figure 7:**
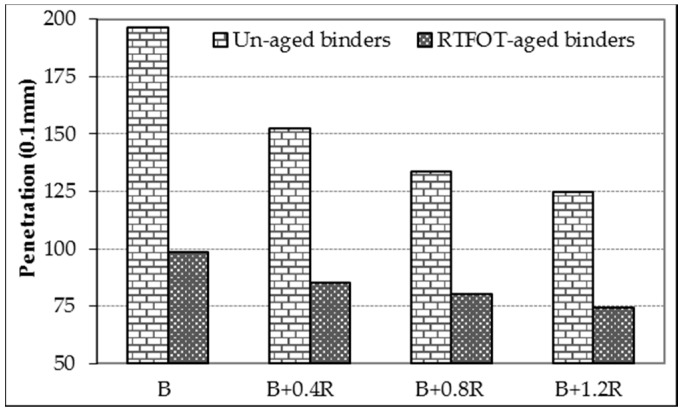
The penetration changing of binders.

**Figure 8:**
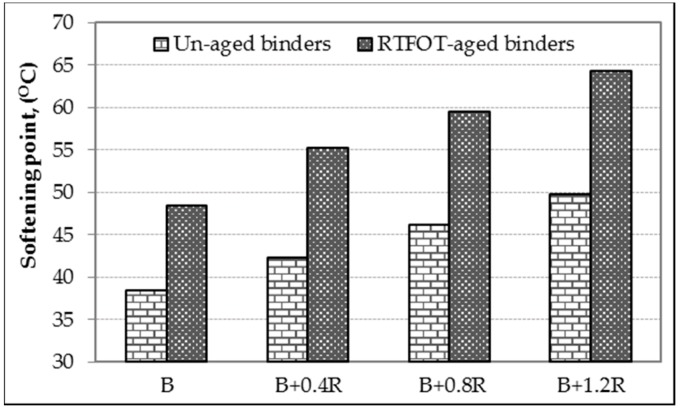
The softening point changing of binders.

**Figure 9:**
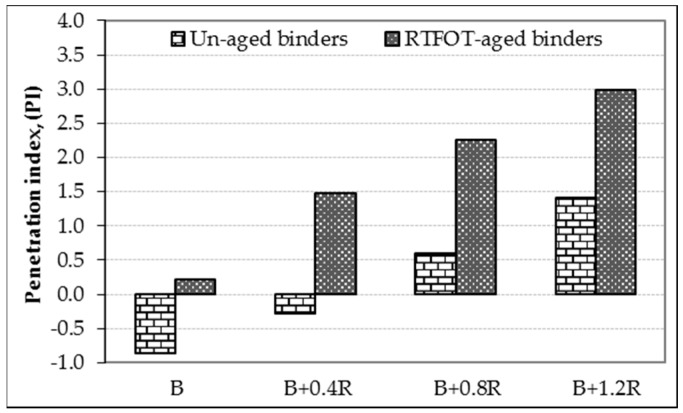
The penetration index (PI) changing of binders.

Basic physical properties of asphalt binders before and after the aging process were determined by conventional bitumen tests, and the results are shown in Table 4. The table also shows the *PI* values which are accepted as a measure of the temperature sensitivity of binders. In addition, the relationship between penetrations, softening points, penetration indexes of the un-aged and RTFOT-aged binders, and the additive amounts in the binder are given in Figure 7, Figure 8, and Figure 9, respectively.

As seen in Table 4 and Figure 7 and Figure 8, when the Elvaloy^®^RET was added to the neat bitumen of 0.4%, 0.8%, and 1.2% by weight, its penetration values decreased by 22.5%, 31.9%, and 36.6% and ductility values by 19.7%, 35.1%, and 42.5%, respectively. On the other hand, the increase of Elvaloy^®^RET content in the blend increased the softening point temperature of modified binder from 38.4 °C to 49.8 °C (i.e., 29.7% increase). The decrease in penetration and ductility as well as the increase in the softening point indicates a significant decrease in the temperature sensitivity of the bitumen and a significant increase in its stiffness. In addition, it was seen that the penetration class of pure B160/220 asphalt binder changed with the Elvaloy^®^RET additive. In this case, it is possible to say that soft consistency binders used in colder climates can be used in warmer regions by adding Elvaloy^®^RET at different rates.

Mass loss results showed that Elvaloy^®^-containing binders exhibited much less mass loss than neat bitumen, and that the loss of mass decreased with increasing Elvaloy^®^ in the blend. This decrease was 20.5%, 48.7%, and 84.6%, respectively, depending on the additive amount. In this case, it can be said that the asphalt binders become less aged or harder with the addition of Elvaloy^®^RET. This also shows that the chemical reaction between bitumen and Elvaloy^®^RET improved the resistance of the modified bitumens to temperature and oxidation.

As seen in Figure 9, when the effect of Elvaloy^®^RET on the *PI* of the binders is examined, it is seen that Elvaloy^®^RET-modified bitumens have much higher *PI* values compared to neat bitumen. This rate of increase was 67.4%, 168.4%, and 264.0%, respectively, depending on the additive amount. This indicates that Elvaloy^®^-modified bitumens with higher *PI* values are more resistant to cracking at low temperatures and permanent deformation at high temperatures [29,46].

When the results of RTFOT-aged binders were examined, it was observed that their penetration values decreased but softening point values increased, as in un-aged binders. In addition, compared to un-aged bitumens, the retained penetrations of the aged binders increased by 50%, 56%, 60%, and 60%; and the softening point values increased by 10.1 °C, 12.9 °C, 13.3 °C, and 14.5 °C, respectively. These results show that the neat and modified bitumens become naturally harder and less fluid after short-term aging, and as a result they are much less sensitive to temperature, especially at high temperatures.

### 3.4. Rotational Viscosity Test

**Figure 10:**
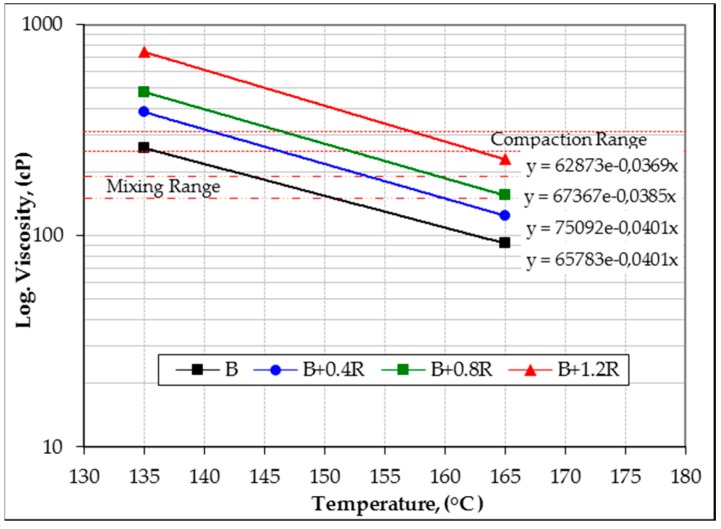
Temperature–viscosity relationship of binders.

The viscosity values of bitumens at high temperatures of 135 °C and 165 °C were determined using a Brookfield viscometer (DV-III). Based on these values, the mixing and compaction temperature ranges of the bitumens for the hot mixtures were determined by means of the temperature–viscosity graph shown in Figure 10. The viscosity values (η), modification indexes (the η value of the modified bitumen was divided by the η value of the pure bitumen) and mixing-compaction temperature ranges of all binders are shown in Table 5.

Figure 10 and Table 5 demonstrate that the viscosity values, which characterize the required flow attributes of the binders to ensure safety in pumping and handling, have increased in parallel with the increase in Elvaloy^®^RET. The increase rates are 48.0%, 84.3%, and 185.0% at 135 °C, and 35.3%, 68.7%, and 150.0% at 165 °C, respectively, compared to pure B 160/220 bitumen. Depending on the viscosity increases, the mixing and compaction temperatures of the modified bitumens increased by 2.4%, 4.7%, and 8.1% and 2.2%, 4.4%, and 7.7%, respectively. These results showed that the bitumen hardness increased significantly due to the increase in the amount of Elvaloy^®^ in the bitumen-additive blend.

### 3.5. Dynamic Shear Rheometer Test

**Figure 11:**
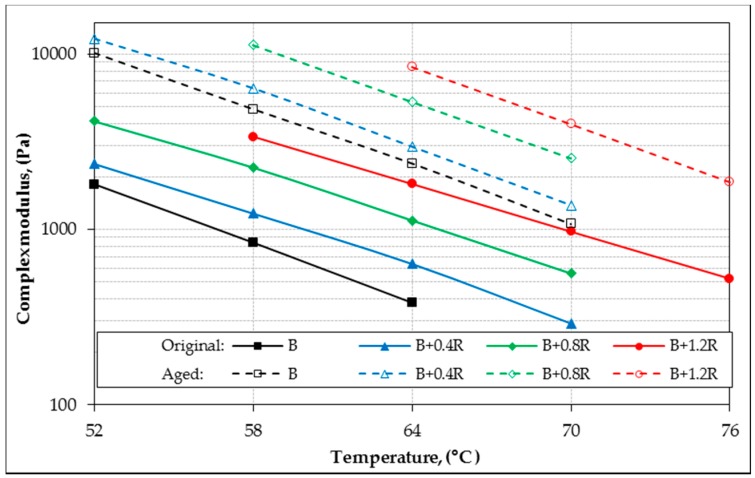
The complex modulus (G*) and temperature relationship of binders.

**Figure 12:**
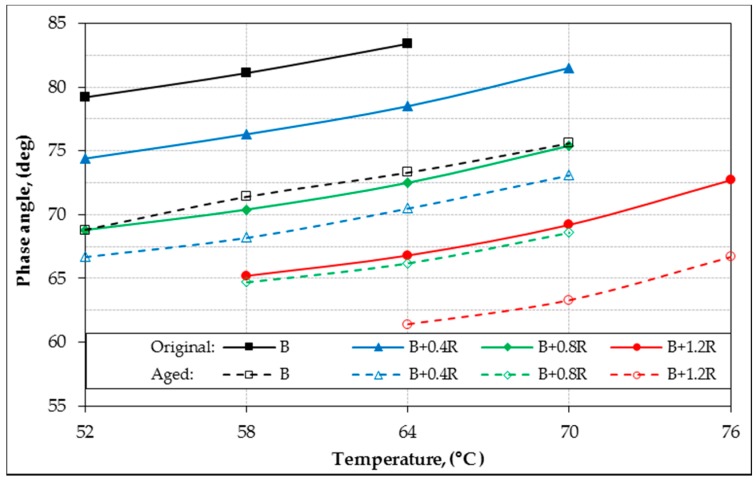
The phase angle (δ) and temperature relationship of binders.

**Figure 13:**
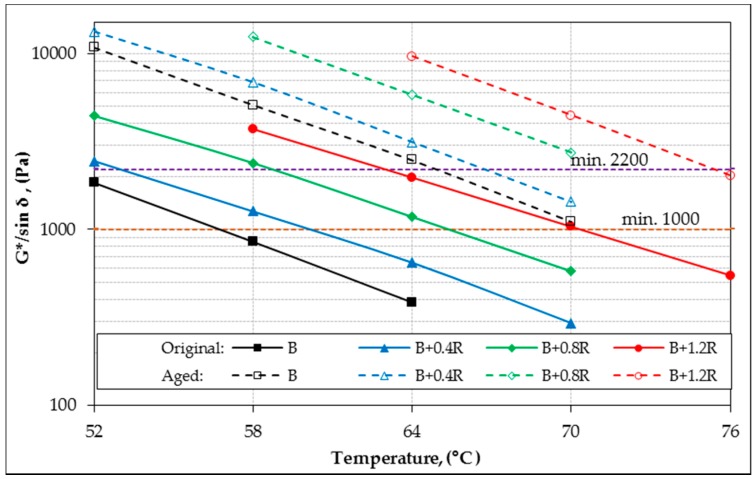
The G*/sin δ and temperature relationship of binders.

**Figure 14:**
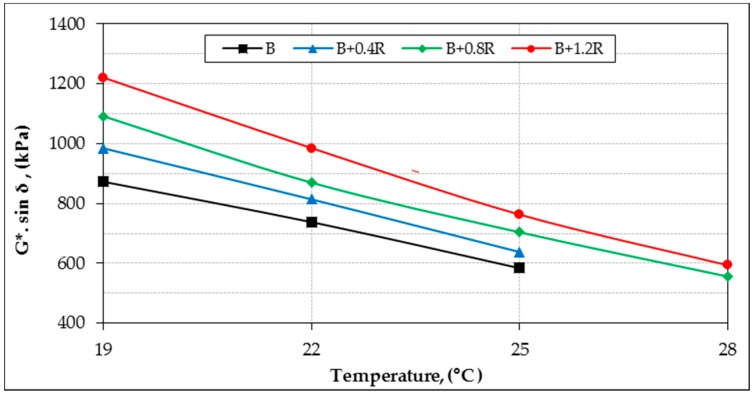
The G*.sin δ and temperature relationship of binders.

In the study, the rheological properties (G*, δ) of all bitumens at intermediate and high service temperatures were identified by DSR device. According to the rutting parameters (G*/sin δ) of bitumens, performance grades were determined for high temperatures. In addition, fatigue resistance parameters (G*.sin δ) of binders were also evaluated for intermediate temperatures. The original (unaged) and aged DSR results of all binders for different temperatures are shown in Table 6. For all binders, the relationship between temperature with G *, δ, G*/sin δ and G*.sin δ are shown in Figure 11, Figure 12, Figure 13, and Figure 14, respectively.

Table 6 and Figure 11 show that the G* values have significantly increased in both aged and un-aged bitumens due to the increase of Elvaloy^®^. However, as seen in Figure 12, increases in the Elvaloy^®^ ratio caused a decrease in the phase angle values of the bitumens. This result shows that Elvaloy^®^-modified bitumens behave as a more elastic solid. Increases in the complex modulus (G*) value, which is a measure of the total deformation resistance of a binder exposed to shear stresses, and decreases in the phase angle (δ) value, indicate that Elvaloy^®^-modified binders are much more resistant to permanent deformations. This is because higher G* and lower δ values indicate an increase in permanent deformation resistance.

Figure 13 shows that the rutting parameter (G*/sin δ) values of Elvaloy^®^-modified bitumens were significantly increased compared to the neat bitumen values at the same temperature. When the Superpave limit values were evaluated together, it was seen that the neat bitumen met the criteria requirements up to 56.5 °C but the high temperature performance level was 52 °C.

Similarly, the temperatures at which Elvaloy^®^RET-modified bitumens met the limits were determined as 60 °C, 65.5 °C, and 70.5 °C, respectively. According to these values, the performance temperatures of the modified binders were determined as 58 °C, 64 °C, and 70 °C, respectively. As a result, it was determined that the high temperature performance classes of all bitumens according to Superpave criteria were PG 52-Y, PG 58-Y, PG 64-Y, and PG 70-Y respectively. Furthermore, according to the neat bitumen, 6 °C, 12 °C, and 18 °C increments occurred at the performance temperatures of the modified bitumens, respectively. This means that when modified with 1.2% Elvaloy^®^RET, a pure bitumen, which should normally be used at a temperature of up to 52 °C, can be used at a temperature of up to 70 °C.

Furthermore, as seen in Figure 14, the fatigue resistance parameter values of all binders (G*.sin δ) remained below the limit value of 5000 kPa. This indicates that the fatigue crack resistance of the modified bitumens is sufficient at intermediate temperatures. These results imply that binder-induced fatigue cracks may not occur in an asphalt pavement exposed to repeated traffic loads.

Based on the DSR test results, it was seen that the addition of the Elvaloy^®^ increased the stiffness of bitumen and reduced the temperature sensitivity, but made it an elastic solid. As a result, it was determined that Elvaloy^®^RET modified bitumens had higher permanent deformation resistance at higher temperatures.

### 3.6. Bending Beam Rheometer Test

**Figure 15:**
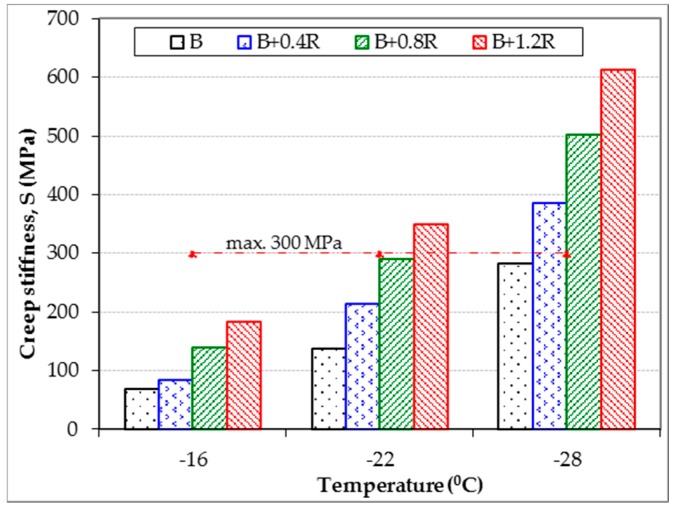
Effects of Elvaloy^®^RET on the creep stiffness of B 160/220.

**Figure 16:**
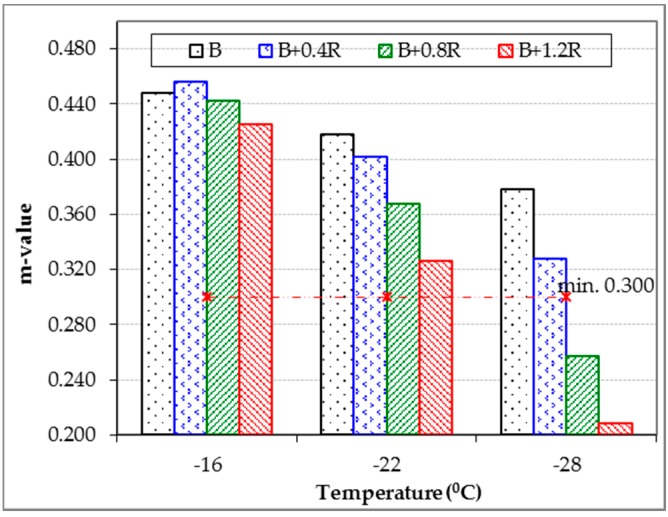
Effects of Elvaloy^®^RET on the *m*-value of B 160/220.

Creep stiffness (S) and creep ratio (m-value) values, which are considered as performance parameters of bitumens exposed to very low temperatures, were obtained by using a BBR tester. The BBR results of the neat and modified bitumens at different temperatures were obtained 60 s after the start of the test, and these results are given in Table 7. In addition, the relationships of these parameters with temperature are given in Figure 15 and Figure 16.

As seen from Table 7 and Figure 15, the stiffness of modified bitumens increased at low temperatures due to the increase in the amount of Elvaloy^®^RET. This means that bitumens containing Elvaloy^®^RET exhibit a more rigid behavior. However, as seen in Figure 16, the m-value values decreased with the increase of the additive rate. This means that there is a relative increase in the hardening speed of the Elvaloy^®^RET-modified binders exposed to low temperatures. According to this result, it can be stated that cracking at low temperatures may occur in these binders. Tiis is because the rapid change in hardness due to the low temperature can create cracks in the binder under the accumulated thermal stresses.

Low temperature limits of the neat and modified binders according to Superpave specification limits (the creep stiffness is max 300 MPa, the m-value is min 0.3) were obtained as −28.5 °C, −25.0 °C, −22.5 °C, and −20.5 °C, respectively. However, low temperature performance levels of the binders were determined to be −28 °C, −22 °C, −22 °C, and −16 °C, respectively. According to these values, it was determined that the low temperature performance classes of all binders were PG X-28, PG X-22, PG X-22, and PG X-16 respectively. These temperature values show that the Elvaloy^®^RET additive has reduced the low temperature performance of the B 160/220 bitumen from −28 °C to −16 °C. However, the low temperature performance levels of 0.4% and 0.8% Elvaloy^®^RET-modified binders were similar, although they had different hardness and hardening speed.

As a result, when a bitumen containing Elvaloy^®^RET is used at very low temperatures, cracking resistance may be reduced or cracking resistance is increased when used at relatively high temperatures. In fact, this can be seen as a negative situation only when it is evaluated in terms of low temperature, but when examined together with high temperature it will be seen that it is not negative. This is because an asphalt binder which can perform at high performance both at very low temperatures and at very high temperatures has not been produced yet.

### 3.7. Effects of Elvaloy^®^RET on Performance Grading of B 160/220

The effects of the Elvaloy^®^ additive on the low (LT) and high (HT) temperature performance properties and performance grading of the B 160/220 bitumen are given in Table 8.

As can be seen in Table 8, the high and low temperature levels providing the specification limits of the pure B 160/220 bitumen are 56.5 °C and −28.5 °C. However, its high temperature level increased by 6.2%, 15.9%, and 24.8%, respectively, in parallel to the increase in Elvaloy^®^RET. Due to these increases, the performance level of bitumen increased from 52 °C to 70 °C. This indicates that the bitumen is severely hardened with Elvaloy^®^RET. However, its low temperature level decreased by 12.3%, 21.1%, and 28.1%. This reduction reduced the low temperature performance level of the bitumen from −28 °C to −16 °C. This can be met as a natural situation because hardening can lead to a reduction in the low temperature crack resistance of the bitumen. When the low and high temperature performance levels of bitumens are evaluated together, it is seen that their performance grades are PG 52-28, PG 58-22, PG 64-22, and PG 70-16 respectively. In this case, it can be said that 1.2% Elvaloy^®^RET can provide a significant benefit to the pavement performance for high service temperatures.

Finally, compared to the neat bitumen, it is possible to conclude that the bitumens modified with the Elvaloy^®^RET perform better at high temperatures, but there is a decrease in their low temperature performance.

## 4. Conclusions

In this study, the effects of reactive terpolymer on the engineering properties of B 160/220 bitumen were investigated, and the following results were obtained.

(1) In the modified bitumen preparation process, when 1.4% or more of Elvaloy^®^RET was added to the bitumen, a gelling occurred in the blend due to the chemical reaction. This result shows that the Elvaloy^®^RET amount, which can be used in bitumen modification, is limited. This limit value was determined as a maximum of 1.4% for B 160/220 bitumen.
(2) The XRD and SEM analysis results indicated that Elvaloy^®^RET interacted with the main macromolecular groups in the bitumen, and Elvaloy^®^RET-modified bitumens behaved as a homogeneous single phase structure as a result of the chemical reaction in the bitumen blend.
(3) According to the results of the conventional test, with the increase of Elvaloy^®^, the stiffness of bitumen increased, the temperature sensitivity decreased, and the penetration class changed. This means that the bitumens modified with Elvaloy^®^RET are more suitable for high service temperatures. The reason for this change is thought to be the chemical reaction between bitumen and Elvaloy^®^RET.
(4) The RTFOT results show that the increase of Elvaloy^®^RET in the mixture results in much less aging of the bitumen. In other words, Elvaloy^®^RET-modified bitumens undergo much less mass loss during mixing and compaction. This shows that the chemical reaction in the mixture increases the resistance of the modified bitumen to temperature and oxidation.
(5) The RV results showed that Elvaloy^®^ increased the viscosity value of pure bitumen by 22% at 135 °C and 33% at 165 °C. This shows that Elvaloy^®^ increases the stiffness of the bitumen and hence its workability temperature.
(6) The DSR results showed that G* values of modified bitumens increased with Elvaloy^®^ additive but δ values decreased. Increases in G* and decreases in δ indicate that the modified bitumens were more resistant to repetitive loads and behaved as a relatively more elastic solid. Higher G* and lower δ values are required for permanent deformation resistance. In this context, it was seen that the pure bitumen, which can be used at a maximum of 59.5 °C in terms of Superpave rutting requirement, can be used up to 70.5 °C with 1.2% Elvaloy^®^. According to the results, it was determined that the high temperature performance classes of bitumens were PG 52-Y, PG 58-Y, PG 64-Y, and PG 70-Y, respectively. This showed that Elvaloy^®^-modified bitumens would be more suitable for use in hot climates.
(7) BBR test findings showed that the hardness of the modified bitumens exposed to low temperatures increased and also the rate of their hardening increased with the application of the load. In fact, it is already natural that a bitumen aged with PAV becomes more rigid when exposed to low temperature, but the increase in hardening speed may be considered negative. However, considering the high and low temperature performances, the performance classes of the bitumens are seen to be PG 52-28, PG 58-22, PG 64-22, and PG 70-16, respectively. Accordingly, it is possible to say that Elvaloy^®^RET can be used at different rates according to different high and low temperature parameters of a region.

As a result, it can be stated that the increase in the amount of Elvaloy^®^RET in the bitumen hardens it and reduces the temperature sensitivity, enhances its elasticity, and increases the fatigue and rutting resistance but decreases its thermal cracking resistance. This indicates that Elvaloy^®^-modified bitumens are more suitable for intermediate and high temperatures. In addition, the strong bitumen–polymer bond formed by the chemical reaction between Elvaloy^®^RET and the bitumen components is thought to improve the performance of the modified bitumen.

## Figures and Tables

**Table 1:** The physical properties of the neat bitumen.

Property	Standard	Specification Limit	B 160/220
Penetration at 25 °C, 100 g, 5 s, 0.1 mm	ASTM D5	160–220	196.4
Softening point, °C	ASTM D36	35–43	38.4
Ductility at 25 °C, 5 cm/min, cm	ASTM D113	>100	114
Flash point, °C	ASTM D92	>220	232
Solubility in trichloroethylene, %	ASTM D2042	>99	99.7
Specific gravity at 25 °C, gr/cm^3^	ASTM D70	1.0–1.1	1.030
Penetration index (PI)	-	-	−0.86

**Table 2:** The physical properties of Elvaloy^®^ 5160 [35].

Composition	Standard	Elvaloy^®^ 5160
Molecular structure	-	Linear
Density, g/cm^3^	ASTM D792	0.95
Physical form	-	Pellet
Melt flow rate, g/10 min	ASTM D1238	12
Melting point, °C	ASTM D3418	80
Maximum Processing Temperature	-	220 °C

**Table 3:** The characteristic properties of PPA.

Properties	PPA
**Chemical Properties**	
Formula	H(PO_3_H)_n_OH
Equivalent H_3_PO_4_ (%)	105
P_2_O_5_ (%)	76.1
**Physical Properties**	
Appearance at 25 °C	Viscous liquid
Specific gravity at 25 °C, gr/cm^3^	1.94
Viscosity at 25 °C, cP	800
Freezing point, °C	16
Boiling point, °C	300

**Table 4:** The physical properties of binders before and after RTFOT process.

Property/Unit	Binder Types			
	B	B + 0.4R	B + 0.8R	B + 1.2R
Penetration at 25 °C, 100 g, 5 s, 0.1 mm	196.4	152.3	133.8	124.5
Softening point, °C	38.4	42.3	46.2	49.8
Ductility at 25 °C, 5 cm/min, cm	114.0	91.5	74.0	65.5
Penetration index (PI)	−0.86	−0.28	0.59	1.41
**After RTFOT**				
Loss on heating, wt%	0.78	0.62	0.40	0.12
Penetration at 25 °C, 100 g, 5 s, 0.1 mm	98.5	85.0	80.1	74.5
Retained penetration, %	50	56	60	60
Softening point, °C	48.5	55.2	59.5	64.3
Change in softening point, °C	10.1	12.9	13.3	14.5
Penetration index (PI)	0.21	1.48	2.25	2.98

**Table 5:** Viscosities and processing temperatures of binders.

Properties	Binder Types			
	B	B + 0.4R	B + 0.8R	B + 1.2R
Viscosity (cP, 135 °C)	260.5	385.5	480.0	742.5
Viscosity (cP, 165 °C)	92.0	124.5	155.2	230.0
Modification index (η_modified_/η_pure_, 135 °C)	1.0	1.48	1.84	2.85
Modification index (η_modified_/η_pure_, 165 °C)	1.0	1.35	1.69	2.50
Mixing temperature range (°C)	145–152	149–155	152–159	157–164
Compaction temperature range (°C)	134–139	137–142	140–145	144–150

**Table 6:** The DSR test results of all binders.

DSR Test Results				
Temperature (°C)	G*/sinδ (kPa) (Specification Limit Min. 1 kPa)			
	B	B + 0.4R	B + 0.8R	B + 1.2R
52	1.843	2.451	4.430	-
58	0.851	1.267	2.388	3.724
64	0.385	0.648	1.179	1.975
70	-	-	0.579	1.038
76	-	-	-	0.549
G*/sin δ (kPa) RTFOT residue (Specification Limit Min. 2.2 kPa)				
52	10.858	13.316	-	-
58	5.114	6.871	12.432	-
64	2.485	3.143	5.839	9.619
70	1.110	1.437	2.737	4.455
76	-	-	-	2.023
G*.sin δ (kPa) PAV Residue (Specification Limit Max. 5000 kPa)				
19	873	985	1092	1220
22	738	814	870	985
25	584	638	704	763
28	-	-	556	594
**High Temperature Performance Grades (PG)**				
	52-Y	58-Y	64-Y	70-Y

**Table 7:** BBR test results of all binders.

BBR Test Results				
Temperature (°C)	m-Value (Specification Limit Min. 0.300)			
	B	B + 0.4R	B + 0.8R	B + 1.2R
−16	0.448	0.456	0.442	0.425
−22	0.418	0.402	0.368	0.326
−28	0.378	0.328	0.257	0.208
**Creep Stiffness (Mpa) (Specification Limit Max. 300 MPa)**				
−16	68.45	84.21	138.45	182.22
−22	138.12	213.47	289.23	348.78
−28	282.19	386.19	502.15	612.63
**Low Temperature Performance Grades (PG)**				
	X-28	X-22	X-22	X-16

**Table 8:** The effects of Elvaloy^®^RET on performance grading of B 160/220 bitumen.

Binder Types	HT (°C)	LT (°C)	HT (°C) Improvement	LT (°C) Improvement	Performance Grading (PG)
B	56.5	−28.5	-	-	PG 52–28
B + 0.4R	60.0	−25.0	3.5	3.5	PG 58–22
B + 0.8R	65.5	−22.5	9.0	6.0	PG 64–22
B + 1.2R	70.5	−20.5	14.0	8.0	PG 70–16
